# Bio-inspired artificial somatic index for reflecting the travel experience of passenger agents under a flexible transportation scenario

**DOI:** 10.1038/s41598-023-44414-x

**Published:** 2023-10-16

**Authors:** Daniel Cabrera-Paniagua, Diego Flores, Rolando Rubilar-Torrealba, Claudio Cubillos

**Affiliations:** 1 Escuela de Ingeniería Informática, Universidad de Valparaíso, Valparaíso, Chile; 2 Departamento de Administración y Economía, Universidad de La Frontera, Temuco, Chile; 3 Escuela de Ingeniería Informática, Pontificia Universidad Católica de Valparaíso, Valparaíso, Chile

**Keywords:** Mathematics and computing, Applied mathematics, Computational science, Computer science

## Abstract

This work analyzes the implementation of an artificial mechanism inspired by a biological somatic marker that ables a passenger agent to both, react to changes in the service, as well as keep said reactions as a memory for future decisions. An artificial mental model was designed, and the passenger agent was implemented as an autonomous decision-making system, where both, the choice of the transport operator and the evaluation of the received service were fully delegated to the system. The evaluation of the service experience is not only based on rational aspects (such as the cost of the trip) but also on subjective aspects related to the satisfaction level derived from the passenger's experience. The experimental scenario considered 10,000 trip requests simulated within an artificial map that emulates characteristics that are usually present in a city, such as vehicular congestion, the unsafety of certain streets, or the benefits of an area with tourist interest. The results show that the option to travel under a transport operator with a touristic profile is a trend. Unlike current cases in the industry, this research work explores the scenario where the passenger can have as a client a trip profile with memory, differentiated from other clients, and can receive more than one trip proposal for the same trip request, according to the different conditions that the passenger is looking for.

## Introduction

Passenger transportation in urban areas remains a topic of international interest. Criteria such as coverage^1^, reliability^2^, and service efficiency^3,4^ have been supplemented and expanded to include additional criteria and restrictions related to the environment, such as CO2 emissions^5,6^, carbon footprint^7,8^, and circular economy practices in the automotive industry^9,10^. With the emergence and use of autonomous vehicles^11,12^ and the increasing focus on passenger travel experience^13,14^, these considerations have become even more critical. At the urban level, conventional public transportation systems have been complemented by flexible transportation options, such as taxi services (flexible in terms of route and timetable, but more expensive), and in general, by different transportation options that can potentially be integrated into multimodal systems (subway, urban elevators, bicycle, among others, according to each city context).

Over the past decade, with the emergence and expansion of flexible passenger transportation based on the use of mobile applications^15,16^, the possibilities of transportation have increased, fundamentally contributing to the availability and coverage of transportation service as well as its sustainable consumption^17^. However, within the context of mobile app-based transportation, there is usually an asymmetry in the passenger–transport operator relationship. While there is flexibility in the service schedule and the choice of pick-up and drop-off points, passengers have limited control over the travel route, which remains at the discretion of the transport operator.

This work investigates the use of a psychosomatic variable for reflecting the travel experience of passenger agents under a simulated flexible transportation scenario. Particularly, both rational and affective decisions factors are considered within a decentralized decision model. The above allows a digital passenger assistant evaluating by itself the different available travel options, and then, selecting the best option according to the passenger preferences and past experience. Thus, the “best travel route” does not correspond to the single travel option offered by a transport operator but is personalized and chosen by each client/passenger.

When a trip request is sent, the passenger agent must choose between travel proposals generated by three types of transport operators: express (provides faster routes), low cost (offers cheaper routes), and tourist profile (delivers routes with better travel experience). During the transportation service, if real-time route replanning is necessary due to accidents, incidents, or road work, the passenger is notified of the updated route. This modification in the initially established travel conditions leads to variations in travel time, cost, distance, and perceived utility level (a subjective measure of satisfaction) for the passenger. In turn, an (artificial) somatic reaction occurs within the passenger agent. This reaction is modulated through an artificial somatic index, which is registered in association with the specific transport operator providing the service. In the next trip request, the passenger agent utilizes information from the artificial somatic index to evaluate whether to select the same transport operator again. It is important to note that each transport operator has an independent artificial somatic index associated with the passenger's experience of their service.

The somatic marker hypothesis, proposed by Antonio Damasio in the 90 s^18^, suggests that individuals experience bodily sensations and changes when making decisions. These sensations and bodily changes accompany and guide decision-making consciously and unconsciously. Somatic markers develop throughout a person’s life, with early stages of life such as childhood and adolescence being particularly significant. In this way, incorporating an artificial mechanism inspired by biological somatic markers provides the passenger agent with the ability to react to service changes and retain these reactions as memories for future decisions. An artificial agent equipped with such a built-in mechanism represents an autonomous decision-making system where both the choice of the transport operator and the evaluation of the received services are fully delegated to the system.

There are different attributes or factors for addressing passenger satisfaction, such as efficiency, security, convenience, amenity^19^, network design, service reliability and professionalism^20^, connectivity, cleanliness, station facilities, operations^21^, to name a few. In the present research work, the evaluation of the service experience is not only based on rational aspects (such as the trip cost), but also subjective aspects related to the on-route passenger's experience, that is, what kind of streets conform the travel route.

There have been some studies proposing the design of artificial somatic markers^22–24^. However, to the best of our knowledge, no previous work has suggested implementing a bio-inspired mechanism based on somatic markers to reflect the passenger's reactions within the flexible passenger transportation domain, understanding the passenger agent as an autonomous decision-making system. Considering all the above, the novelty of our research lies in the following aspects: (1) designing an artificial mental model that incorporates cognitive components and artificial somatic reactions of a passenger agent, (2) developing a bio-inspired mechanism based on somatic marker to reflect the passenger's reaction when facing real-time route replanning, (3) designing an algorithm that uses the mechanism defined in (2) to support decision-making in the flexible passenger transportation domain, (4) defining an experimental scenario for real-time route replanning in the flexible passenger transportation domain, and (5) analyzing the results obtained from one hundred different experimental cycles, each consisting of 100 sequentially received trip request.

The remainder of this work is organized as follows: “Literature review” Section includes a literature review; “Mechanisms description” Section describes the mechanisms in terms of the artificial mental model, mathematical formulation of trip indicators (time, distance, cost, and utility), mechanisms for evaluating travel routes and calculating the artificial somatic index, and the algorithmic for the passenger agent's decision-making; “Scenario description and experimental results” Section presents the scenario description and experimental results; “Discussion” Section discusses the obtained results; finally, “Conclusion” Section concludes the paper and provides suggestions for future research.

## Literature review

Flexible passenger transportation represents a service modality that allows the passenger to define the trip's start time and origin and destination coordinates. Over the past decade, traditional urban taxi services, which relied on telephone calls or face-to-face interactions, have been accompanied by the emergence of flexible transportation services based on mobile applications^25–27^. Examples of such transport operators are^28–30^, which enable passengers to indicate their intention to travel from a specific origin to a destination. The transport operator then links the passenger with an available vehicle whose driver is affiliated with said operator. Since the passenger is human, who requests the service, they usually express their level of conformity with the service received and consider this experience in the next trip request eventually. Several studies have analyzed passenger experiences and travel preferences^31–35^, discussing factors such as passenger loyalty, app-based booking experience, hospitality during the journey, and travel post-booking service.

On the other hand, regarding works related to automated systems in the passenger transportation domain, several studies focus on autonomous vehicles^36–38^, transportation within smart cities^39–41^, and the impacts of mobility services^42,43^, among other things. Specifically in relation to the use of artificial agents in passenger transportation, studies have simulated information availability^44^, optimized passenger evacuation optimization in metro stations^45^, and simulated passenger boarding and alighting spatially confined transportation scenarios^46^. A study by Cabrera-Paniagua et al. (2022) incorporated affective criteria into the decision-making of cognitive agents within the flexible passenger transportation domain. The study focused on the integration of artificial emotions in passenger agents. However, it did not contemplate an experimental scenario with repeated trip requests from the same passenger, the above with the aim to make use of previous travel experiences. Likewise, it did not incorporate the design of any artificial somatic marker. The design and implementation of artificial somatic markers in autonomous decision-making systems have been minimally explored, with applications limited to tasks such as card gambling^22^, theoretical tourism case studies^47^, and stock markets^23,24^.

Considering the aforementioned literature, we observe the availability of technologies aimed to support passenger transportation processes. However, these technologies still require significant human intervention, from trip request submission to the generation of service evaluations that can be retained and considered for future trips. Meanwhile, autonomous vehicle technology primarily focuses on vehicle navigation, rather than representing the interests of human passengers within an automated environment for negotiating and contracting transportation services. The incorporation of affective aspects within autonomous systems based on agents has seen increasing attention in recent years^48,49^. However, to the best of our knowledge, no previous work has suggested implementing a mechanism based on somatic markers to reflect the passenger's reactions within the flexible passenger transportation domain, treating the passenger agent as an autonomous decision-making system.

In this work, given the impossibility of carrying out field tests with an already established passenger transport provider (e.g., an enterprise of app-based ride services) that would like to incorporate the dimension of user preferences within their route planning system, a simulation technique has been used. In addition, the application context of our proposal of user preferences and affective factors model does not consider traditional centralized planning, but rather a decentralized approach, thought of as an open market of transportation providers and demanders (customers). Considering the above, the traditional measures of minimizing the total distance traveled, route time and waiting time (in a centralized single objective function) do not represent the novelty of the proposal. The novelty is outlined on the side of the client who uses a flexible transport system and how his preferences (both rational and emotional in nature) are incorporated into a digital assistant (artificial passenger agent) that is capable of negotiating the best route with one or more transport operators, where the idea of “best route” is personalized to each client/passenger. Therefore, what is interesting to verify is that the conceived model is capable of capturing this complexity and variability in terms of preferences and types of users.

## Mechanisms description

### Artificial mental model

**Figure 1:**
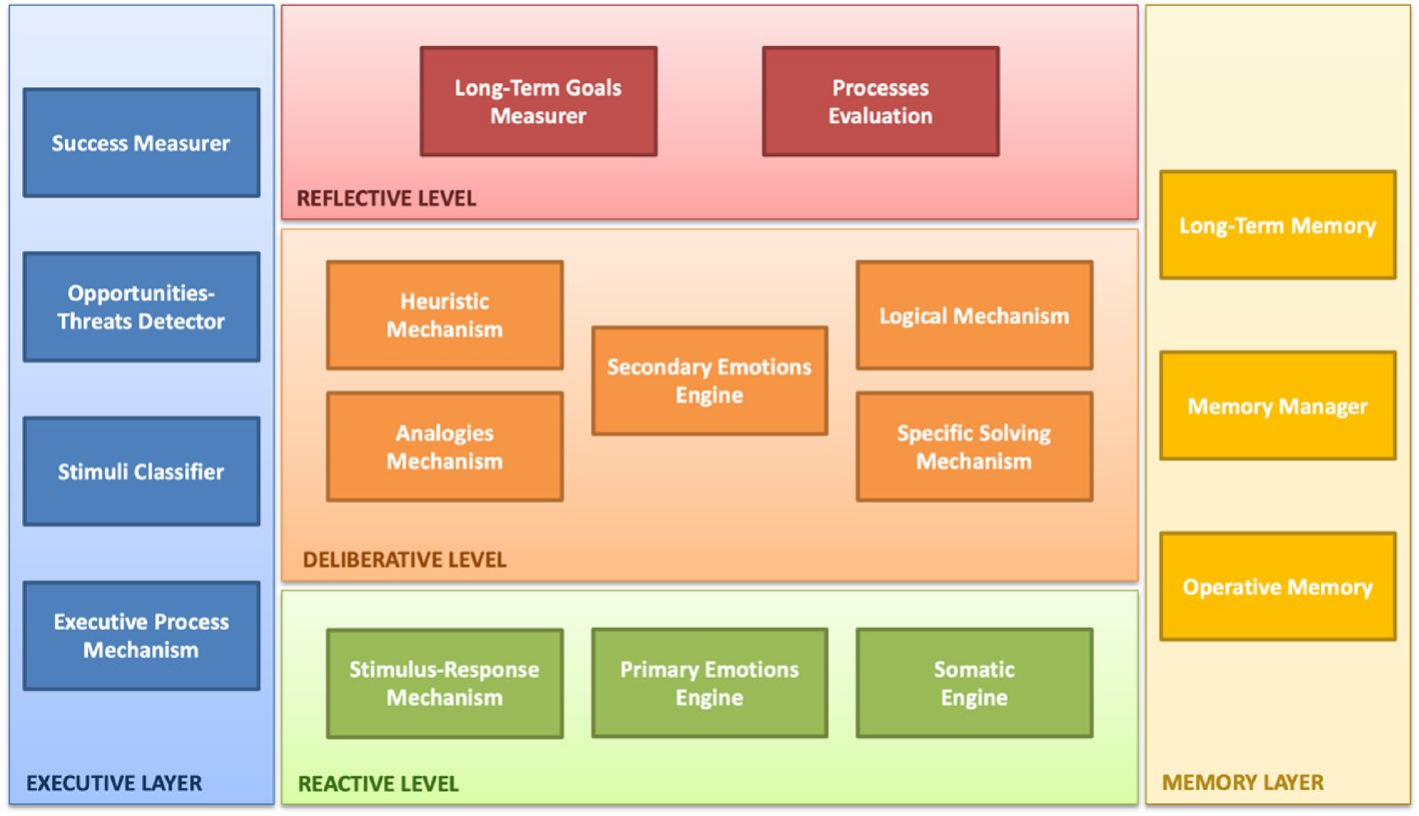
Artificial mental model (source: own elaboration).

Following Minsky's mental model of general purposes^50^ as a general guide, Fig. 1 shows an approach of an artificial mental model for the passenger agent, composed of three levels—Reactive, Deliberative, and Reflective—and two layers—Executive and Memory.

*Reactive Level*: This level represents the first level of response to internal and external stimuli. The "*Stimulus–Response Mechanism*" is directly associated with actions such as "If … then Do." Additionally, the component "*Primary Emotions Engine*" controls the valuation and status of the entity's primary emotions.

*Deliberative Level*: This level addresses stimuli and situations that require more sophisticated responses. Its components are as follows:

- *Analogies Mechanism* This component looks for analogies between the current problem or situation and past successful mechanisms used for problem-solving and decision-making.
- *Heuristic Mechanism* This component allows the use of ad hoc methods or experience-based methods to address specific situations.
- *Logical Mechanism* This component applies logical rules to problem-solving and decision-making.
- *Specific Solving Mechanism* This component represents all procedures the entity employs to deal with situations and respond to stimuli that cannot be classified under the above components.
- *Secondary Emotions Engine* This component combines knowledge, experience, feelings, primary emotions, and somatic reactions.

*Reflective Level*: This level enables the entity to think and reflect on its decision-making processes and the accomplishment of its goals. In addition, it is possible to reflect on how its internal processes are performed, including the use of heuristics, logical procedures, and analogies mechanism.

*Executive Layer*: This layer has mechanisms to classify stimuli based on their origin, level of intensity, duration, etc. It also identifies opportunities and threats in the environment that may affect goal achievement, and measures success in decision-making. The component called "*Executive Process Mechanism*" is enabled to control and monitor the implementation of each decision.

*Memory Layer*: This layer includes the "*Memory Manager*" component, which is responsible for storing and retrieving information required for the current action (Operative Memory) as well as information related to past events, situations, and decisions (Long-Term Memory).

### Mathematical formulation

This work considers a simulated artificial city comprising four types of streets: normal, unsafe, congested, and tourist. For the purposes of this research work, the travel time corresponds to the simple sum of the required transit time for each of the streets that make up a travel route:

1 $$time_{t} = \mathop \sum \limits_{i = 1}^{n} nStreet_{i,t} \cdot nt + \mathop \sum \limits_{j = 1}^{u} uStreet_{j,t} \cdot ut + \mathop \sum \limits_{k = 1}^{c} cStreet_{k,t} \cdot ct + \mathop \sum \limits_{l = 1}^{t} tStreet_{l,t} \cdot tt,$$

where *nStreet*_*i,t,*_* uStreet*_*j,t*_*, cStreet*_*k,t*_*,* and *tStreet*_*l,t*_ represent the trip made from street *i* in iteration *t*, corresponding to a normal, unsafe, congested, and tourist street, respectively. On the other hand, the parameters *nt*, *ut*, *ct*, and *tt*, represent the time factors of a normal, unsafe, congested, and tourist street, respectively.

The travel distance corresponds to the simple sum of the required transit distance for each of the streets that make up a travel route:

2 $$distance_{t} = \mathop \sum \limits_{i = 1}^{n} nStreet_{i,t} \cdot nd + \mathop \sum \limits_{j = 1}^{u} uStreet_{j,t} \cdot ud + \mathop \sum \limits_{k = 1}^{c} cStreet_{k,t} \cdot cd + \mathop \sum \limits_{l = 1}^{t} tStreet_{l,t} \cdot td$$

where the parameters *nd*, *ud*, *cd*, and *td*, represent the distance factors of a normal, unsafe, congested, and tourist street, respectively, similar to the definition of Eq. (1), considering the factor of distance instead of time.

On the other hand, the cost of travel is determined according to Eq. (3):

3 $$cost_{t} = veCost_{t} \cdot vtf + time_{t} \cdot tfc + distance_{t} \cdot dfc,$$

where veCost_t_ corresponds to the operational cost associated with a specific type of vehicle (e.g., city car, luxury car) considered in the trip *t*; *time*_*t*_ corresponds to the travel time of the trip *t* calculated according to Eq. (1); *distance*_*t*_ corresponds to the route distance from an origin point to a destination point in the trip *t* defined in Eq. (2). Parameters *vtf*, *tfc,* and *dfc* correspond to the cost impact of a specific vehicle-type choice, the cost associated with the time spent in transportation, and the cost per unit of distance traveled, respectively.

Meanwhile, the utility derived from travel is determined according to Eq. (4):

4 $$utility_{t} = \mathop \sum \limits_{i = 1}^{n} nStreet_{i,t} \cdot nu + \mathop \sum \limits_{j = 1}^{u} uStreet_{j,t} \cdot uu + \mathop \sum \limits_{k = 1}^{c} cStreet_{k,t} \cdot cu + \mathop \sum \limits_{l = 1}^{t} tStreet_{l,t} \cdot tu,$$

where *nu*, *uu*, *cu*, and *tu* represent the utility factor of a normal, unsafe, congested, and tourist street, respectively, which is the utility derived from traveling a block of this type of street. It is worth mentioning that utility is conceived as a subjective measure of each passenger agent that influences the decision-making process of which type of service to use and tries to be a simile of the way in which human beings make their decisions, based on their experience and stimuli.

When a passenger agent sends a trip request, they may receive several travel proposals, which are evaluated independently by calculating a score, as shown in Eq. (5):

5 $$score_{to,t} = time_{to,t} \cdot ti + cost_{to,t} \cdot ci + utility_{to,t} \cdot ui + somaticIndex_{to,t} \cdot sii$$

where *score*_*to,t*_ represents the score associated with the travel proposal received from the transport operator *to* in the trip *t*; furthermore, *somaticIndex*_*to,t*_, corresponds to an index proposed in this research that links the somatic reaction derived from the transport experience with the quantification of that experience. In this case, *ti*, *ci*, *ui*, and *sii* correspond to parameters representing the weight of time, cost, utility level, and somatic index, respectively.

Concerning the *somaticIndex* variable, a positive value represents a good level of travel experiences of a passenger with the transport operator *to*. Conversely, a negative value represents a poor level of travel experiences of a passenger with the transport operator *to*. It is important to mention that for each passenger-transport operator relationship, there is an independent *somaticIndex* variable that represents said relationship. Likewise, the initial *somaticIndex* value is zero (i.e., a neutral value) and varies over time according to how the passenger evaluates the travel experience derived from transport operator service.

As mentioned in previous sections, this work suggests the incorporation of a somatic index as a mechanism for evaluating and registering the passenger travel experience associated with different transport operators. Particularly, the *somaticReaction* variable allows determining the intensity of the reaction experienced by the passenger after being notified that it is necessary to replan (in real-time) the travel to the destination point. In this sense, *somaticReaction* is calculated according to Eq. (6):

6 $$somaticReaction_{tot} = \tan^{ - 1} \left[ {\Delta time_{tot} \cdot itv + \Delta cost_{tot} \cdot icv + \Delta utility_{tot} \cdot iuv} \right] \pm rand\left[ { - 0.5 , 0.5} \right] \cdot irf$$

where *Δtime*_*to*_, *Δcost*_*to*_, and *Δutility*_*to*_ correspond to the variation between the original travel and the updated travel time, cost, and utility, respectively, associated with the travel proposal received from the transport operator *to* in the trip *t*. On the other hand, the parameters *itv*, *icv*, *iuv*, and *irf* represent the weight of time, cost, utility, and random effect, respectively. For this research, the random effect is uniformly distributed between [− 0.5, 0.5], a value that can be modified according to the specific requirement of the application.

The calculation of the *somaticReaction* variable suggests the use of a smoothing function with the idea of smoothing extreme values (of very high positive intensity or very low negative intensity), such as a logistic, sigmoid, or arctangent function, among others. For this research, the arctangent function was used for calculation simplicity. However, it can be replaced by a more general function depending on the application. Likewise, the incorporation of a random value allows granting a degree of flexibility in the *somaticReaction* value, understanding that exposure of a person to the same stimulus (or result) over time does not guarantee the same somatic reaction. It should be noted that the Somatic reaction is associated with the transport operator *to*.

**Figure 2:**
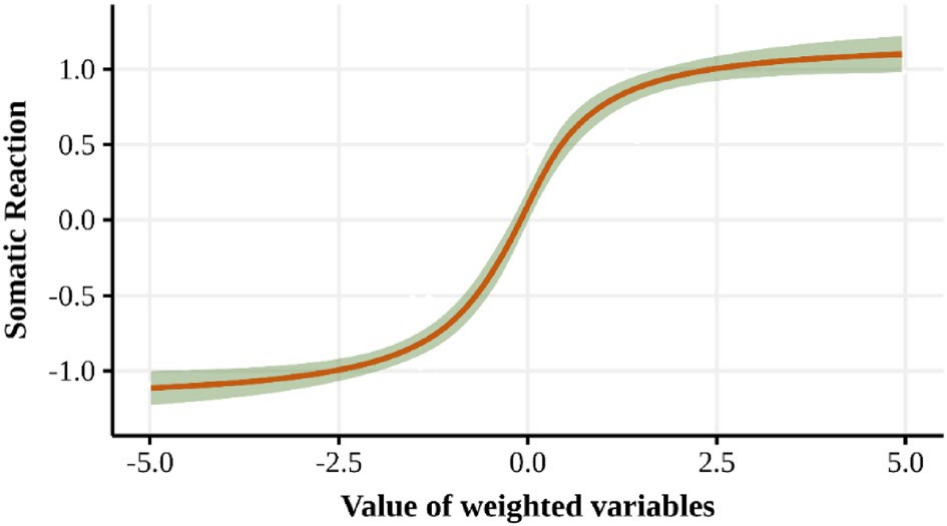
Example of somatic reaction behavior [source: own elaboration].

Below we show the example of somatic reaction behavior for synthetic data in Fig. 2. In this graph, we can see that as the values become more extreme, the value of the reaction tends to stabilize, which allows us to control the overall agent behavior. In addition, confidence bands are shown which correspond to the randomness effect described in Eq. (6).

The *somaticIndex* is defined according to Eq. (7):

7 $$somaticIndex_{to} = siw \cdot somaticIndex_{to} + \left( {1 - siw} \right) \cdot somaticReaction_{tot}$$

where *somaticIndex*_*to*_ initially in zero, registers the passenger's experience regarding the service of a transport operator *to* and *siw* corresponds to the weight of the previous somatic index in the modeling. Somatic index modeling is characterized by an autoregressive effect, in other words, the effect of the previous somatic index affects the observed value of the current somatic index. The modeling proposed in this initial work considers an equivalent weight between the value of the previous somatic index and the somatic reaction, which allows us to conceptualize a memory of the artificial agent about its previous experiences.

Regarding the *somaticIndex*_*to*_ valuation, this research considers assigning an equivalent connotation both to the memory of the "passenger agent—transport operator" relationship and the most recent reaction experienced by the passenger agent prior to the service received from transport operator *to*. In relation to the somatic marker hypothesis, this last experienced reaction by the passenger agent can be associated with a “somatic reward” or a “somatic punishment,” which correspond to positive or negative sensations that may linger in the working memory of the autonomous agent^47^. These sensations can emulate a sense of well-being, regret, or discomfort, and they influence the decision-making process. In the case of this work, the *somaticReaction* variable acts as an artificial somatic reward or punishment based on the assessment derived from the most recent experience.

Algorithm 1 describes the general trip request process of a passenger agent. All the steps contained in the algorithm are associated with the passenger agent. The algorithm begins with the definition of the trip's origin and the destination points. The passenger agent then submits their request. The algorithm utilizes the route determination algorithm designed and presented by^51^. Upon receiving travel proposals, the passenger agent calculates the travel time, distance, cost, and utility for each route. Each travel proposal is assigned a score, and the proposals with the highest score are chosen. The passenger agent then activates the service provision with the corresponding transport operator. Subsequently, the passenger agent receives route information, where if said information corresponds to a replanning notification, the agent obtains the new route to follow. From this new route, the passenger agent recalculates the travel metrics, which comprise the travel time, distance, cost, and utility derived from the route. Once the travel metrics have been updated, the passenger agent experiences an artificial somatic reaction, which, in turn, affects the update of the *somaticIndex*, generating a record in the working memory for the next decision and a record in the long-term memory.

**Figure:**
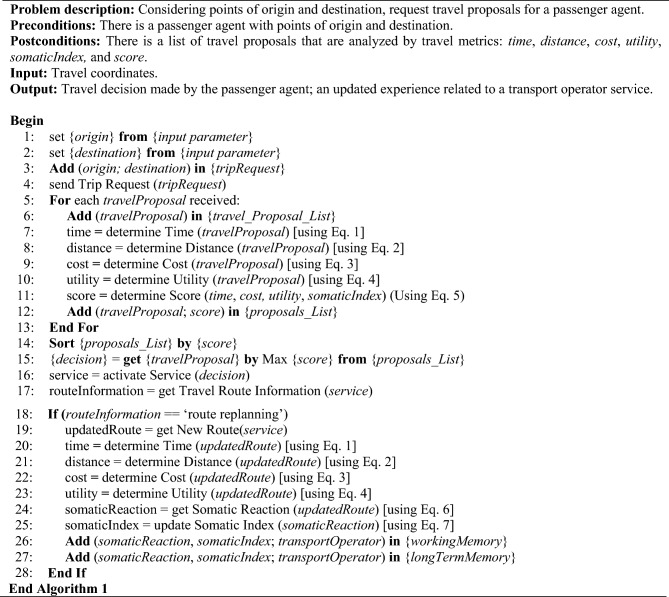
**Algorithm 1.** General trip request process of a passenger agent.

Regarding algorithm 1 and the artificial mental model presented in Fig. 1, steps 1, 2, and 4 are associated with an executive process mechanism (within the executive layer). Meanwhile, steps 3 and 6 are associated with the memory layer, specifically, the operative memory (of the passenger agent). On the other hand, steps 7–11 are specific solving mechanisms since they correspond to mechanisms for calculating travel metrics. Both the travel proposal and its score are recorded in the working memory. Meanwhile, steps 14–17 are associated with the deliberative level since they allow the passenger agent to decide on the transport operator and operationalize said decision. In addition, faced with a real-time route replanning (logical evaluation of step 18 of the algorithm), steps 19–23 associated with calculating travel metrics are performed (that is, at a deliberative level).

The primary effect derived from the route change corresponds to the somatic reaction experienced by the passenger agent and is obtained in step 24 (at the reactive level of the artificial mental model, through the Somatic Engine component). Meanwhile, the subsequent effect of this somatic reaction is obtained in step 25 (at the deliberative level of the artificial mental model, through the Secondary Emotions Engine component), and is reflected by using an index (at the level of secondary emotions) representing the passenger's experience with the service received from a specific transport operator.

## Scenario description and experimental results

### Scenario description

This research work considers a simulated artificial city, represented by a 30 × 30 matrix, where four types of streets are present: normal, unsafe, congested, and tourist. The proportion of each kind of street corresponds to 25% of the total streets, and their distribution within the matrix is randomly determined. A normal street represents a neutral state in terms of travel time and the sensation it evokes in people. An unsafe street has characteristics that may represent a risk or danger to those who pass through it (e.g., poor lighting, presence of blind walls, abandoned sectors), evoking negative sensations in people. Meanwhile, a congested street requires more time to travel and leads to a lower level of satisfaction. Finally, a tourist street also requires more time to travel, but it elicits pleasant sensations. A street is considered to have a single prevailing characteristic (i.e., it can belong to only one of the four previously mentioned types). Likewise, a travel route can contemplate the passage through streets of different types.

The scenario is based on the execution of Algorithm 1 by the passenger agent, who requests to be transported from coordinate (1,1) to coordinate (30,30). Then, the passenger receives three different travel proposals: one from an “express” transport operator (generating faster routes), one from a “low cost” transport operator (generating cheaper routes), and one from a “touristic” transport operator (providing routes with a better travel experience). The passenger agent selects one of the travel proposals. The experimental scenario considers route replanning is always required, triggering a somatic reaction in the passenger agent. The *somaticIndex* specifically associated with the transport operator offering the service is then updated. At the end of the execution of Algorithm 1, the passenger agent has a new travel experience provided by a particular transport operator (whose effect is registered in the respective *somaticIndex* variable).

Algorithm 1 is executed 100 times sequentially by the passenger agent, with the passenger agent requesting transportation 100 successive times from the origin to the destination. Thus, the somatic reactions of the passenger agent are successively transferred from execution to execution, as well as the update of the *somaticIndex* variable associated with the transport operator that provided the last service received. The set of 100 execution times of Algorithm 1 is called "Cycle." The experimental results of this work summarize the execution of 100 independent cycles.

On the other hand, Table 1 shows the general parameters used in the different equations presented previously. The "Time" column is associated with Eq. (1), where time is measured in "time units." The time required to pass through a normal (nt) or unsafe (ut) street is 1 (time unit). Meanwhile, a congested street (ct) and a tourist street (tt) require 3 and 2 units of time, respectively. The “Distance” column is associated with Eq. (2), where the distance traveled on any types of streets will always 1 unit of distance. The "Cost" column is associated with Eq. (3), where the first variable veCost represents an initial operational cost associated with the specific type of vehicle that the transport operator has suggested for the service. Meanwhile, vtf, dfc, and tfc represent the level of importance given to the cost of the vehicle (0.1), the distance traveled on a trip (0.5), and the time spent on the route (0.4), respectively. The “Utility” column is associated with Eq. (4), where the utility derived from transit through a normal (nu), unsafe (uu), congested (cu), and tourist (tu) street is 1, 0.2, 0.5, and 3 utility units, respectively. Meanwhile, the “Score” column is associated with Eq. (5), where travel time, cost, utility, and *somaticIndex* have an importance level of 0.25. Finally, the “somR” column is associated with Eq. (6), where an equivalent level of importance of 0.3 is proposed for the variations of time (itv), cost (icv), and utility (iuv). To give a degree of variability in the *somaticReaction*, a random value has an importance level of 0.1.

**Table 1:** General parameters for all the experimental cycles.

Time (Eq. 1)		Value		Distance (Eq. 2)		Value		Cost (Eq. 3)		Value	Utility (Eq. 4)		Value		Score (Eq. 5)		Value		somR (Eq. 6)		Value	
nt	1		nd		1		veCost		Rand[0,10]			nu		1		ti		0.25		itv		0.3
ut	1		ud		1		vtf		0.1			uu		0.2		ci		0.25		icv		0.3
ct	3		cd		1		dfc		0.5			cu		0.5		ui		0.25		iuv		0.3
tt	2		td		1		tfc		0.4			tu		3		sii		0.25		irf		0.1

Given that the present research work uses synthetic data, for the calculation of the score it has been decided to grant equivalent weights to time, cost, utility, and *somaticIndex*. In the same way, for the calculation of the *somaticReaction* variable, an equivalent weight has been given to the variation of time, cost, and utility, respectively.

### Experimental results

The results of all the experiments for the dimensions “Time,” “Cost,” and “Utility” are summarized in Table 2. It can be seen that the time used to travel from the initial point to the endpoint for the Express service takes an average of 83.37 time units, with a standard deviation of 3.31. The minimum and maximum values correspond to 77.00 and 97.00, respectively, which indicates that the data distribution is skewed to the “left.” Similarly, in the case of the Low Cost and Tourist service, we observe an average value of 84.35, and 89.93, respectively, and a standard deviation of 1.45, 2.59. The above suggests that it is more likely to find observations smaller than larger, in relation to the mean value.

**Table 2:** General descriptive statistics.

Type of service	Dimension	Mean	Standard deviation	Minimum	Maximum
Express	Time	83.37	3.31	77.00	97.00
	Cost	63.93	1.51	61.21	69.28
	Utility	61.57	5.11	41.10	75.00
Low Cost	Time	84.35	3.54	75.00	93.00
	Cost	63.88	1.45	60.18	66.96
	Utility	61.10	5.57	37.70	78.00
Tourist	Time	89.93	4.99	77.00	110.00
	Cost	67.65	2.59	61.4	79.89
	Utility	70.75	6.83	51.30	101.00

In the case of the "Cost" dimension, it is observed a total of 63.93, 63.88, and 67.65 monetary units were spent on average with a level and variability of 1.51, 1.45, and 2.59, considering its standard deviation for Express, Low Cost, and Tourist service, respectively. Similarly, for the time spent, a greater concentration of values can be observed to the "left," although more pronounced, showing the existence of extreme values in costs, lower than the mean value.

The case of the "Utility" dimension shows a total of useful centered at 61.57, 61.10, and 70.75 with a variability of 5.11, 5.57, and 6.83, measured by its standard deviation for Express, Low Cost, and Tourist service, respectively.

**Figure 3:**
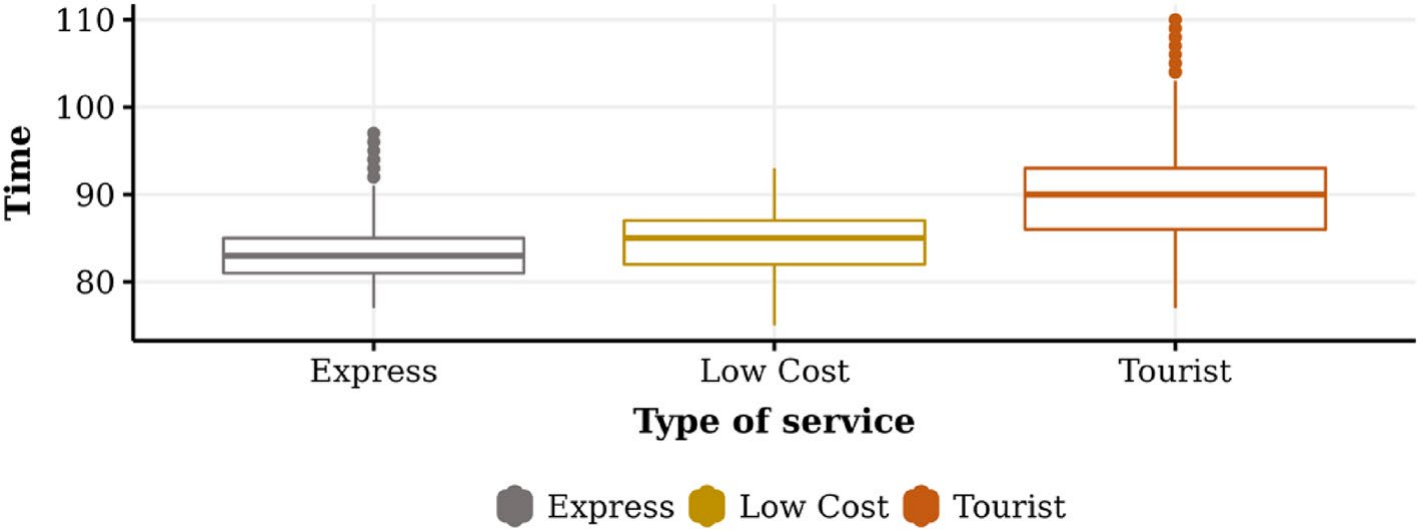
Average of time for each transport operator profile.

**Figure 4:**
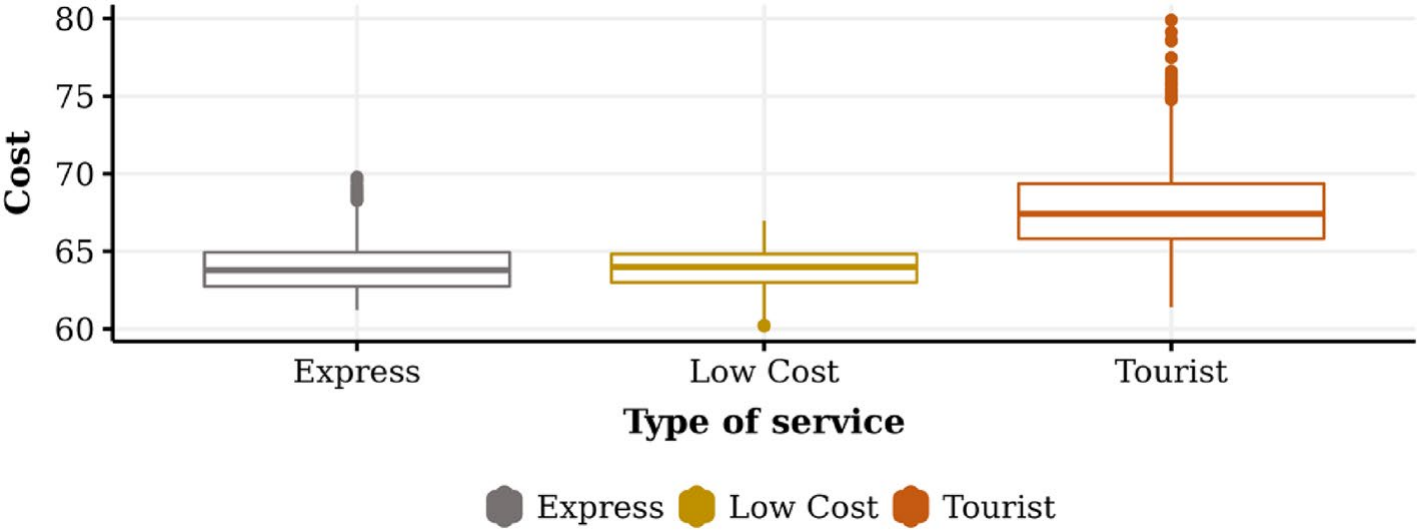
Average of cost for each transport operator profile.

**Figure 5:**
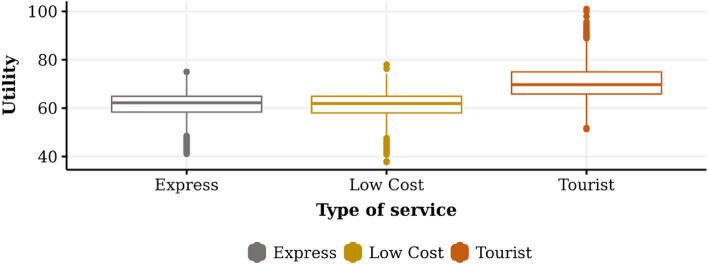
Average of utility for each transport operator profile.

Figures 3, 4, and 5 graphically show the behavior of the experimental results related to time, cost, and utility, respectively. Figure 3 shows the touristic profile transport operator is the one that spends the longest time on its journey and the highest level of variability in its results, which is consistent with the search for routes that deliver higher levels of satisfaction or well-being. The low-cost profile transport operator has less travel time than the tourist company and less variability. Finally, the express profile transport operator is the one that shows the lowest average level of time spent and the lowest variability compared to the previous cases.

Figure 4 shows that the tourist profile transport operator has a higher level of cost and variability. The low cost and express transport operators present a lower level of total costs and less variability in the tourist profile. A lower level of variability is observed in the low-cost case when compared to express, although they are similar with respect to its mean values. The explanation for this phenomenon, in which no major differences were detected concerning cost, is given by experimentation parameters related to the factors of time, vehicle type, and distance that influenced the cost of the service. In this sense, the low-cost service, which seeks routes with shorter distances and cheaper vehicles, can be compared with the express service because the latter seeks routes with a shorter arrival time, reducing the time costs of the service.

Figure 5 shows the level of utility experienced by the passenger agent in the experimental scenario. In the case of the touristic transport operator, it is possible to observe a higher level of average utility and a higher level of variability, finding an important group of values considered extreme. Meanwhile, the utility of the low-cost and express transport operators has similar levels of satisfaction in mean and standard deviation, with no significant differences between them.

Table 3 shows the use levels of the different services available. The touristic transport operator is predominant with a level of use of 63.3% on average, considering the 100 experimental cycles with 100 trips each. The express transport operator obtained a level of use of 26.1%, while the low-cost transport operator obtained 10.6%. It can also be seen that the touristic transport operator presents the highest level of variability measured by its standard deviation, followed by the express and the low-cost transport operators.

**Table 3:** Usage levels for each type of transport operator.

Transport operator	Mean (%)	Standard deviation (%)	Minimum (%)	Maximum (%)
Express	26.1	4.2	17.0	37.0
Low cost	10.6	2.9	4.0	18.0
Touristic	63.3	5.1	49.0	74.0

On the other hand, the choice model between the three types of transport services (choice of transport services) for a period t is defined according to Eq. (8):

8 $$CTS_{t} = f\left( {somaticIndex_{t,lc} ,somaticIndex_{t,exp} ,somaticIndex_{t,tou} } \right),$$

where *somaticIndex*_*t,lc*_*, somaticIndex*_*t,exp*_, *somaticIndex*_*t,tou*_ correspond to the *somaticIndex* of the low cost, express, and touristic service in period t, respectively.

To model the choice of a specific service we propose a Multinomial Logit model:

9 $$\Pr \left( {Y_{i} = j} \right) = \frac{{e^{{\beta_{j} x_{i} }} }}{{\mathop \sum \nolimits_{k = 1}^{3} e^{{\beta_{k} x_{i} }} }}, j = 1, 2, 3,$$

where *Pr*(*Y*_*i*_ = *j*) corresponds to the probability that the passenger agent chooses the service *j*; *x*_*i*_ represents the somatic index variable associated with each transport operator, which is low cost, express, and touristic artificial reactions; and *β* corresponds to the vector of parameters to be estimated for each of the possible choices. The model assumes that the somatic reactions of the different contracted services conditionate the service choice to be taken at the next opportunity. For this research, we are going to assume complete independence in the selection of travel routes, meaning that a route used by a particular service is not correlated with another type of service. In this sense, this assumption, although unrealistic in the real world, allows us to explore selection alternatives through the use of synthetic data and a potential future line of research with the implementation and use of empirical data. This model provides a concrete tool for estimating the parameters of artificial agents, based on behavior that can be observed in the real world, allowing artificial agents to behave similarly to the behavior and decisions of human beings. The regression results of synthetic data are shown in Table 4.

**Table 4:** Multinomial Logit model estimation.

3-categories of artificial agent^a^	$\beta$	Std. Error	95% confidence interval	
			Lower bound	Upper bound
Tourist agent				
Low cost reaction	0.029	0.065	− 0.098	0.157
Express reaction	− 0.138*	0.072	− 0.279	0.003
Tourist reaction	0.261***	0.057	0.149	0.372
Constant	0.771***	0.027	0.717	0.825
Low cost agent				
Low cost reaction	− 0.039	0.083	− 0.203	0.124
Express reaction	− 0.087	0.093	− 0.269	0.095
Tourist reaction	0.132*	0.729	− 0.011	0.275
Constant	− 0.620***	0.035	− 0.689	− 0.551

Number of replicates 10,000.

^a^The reference category Express.

***p < 0.01, **p < 0.05, *p < 0.1.

For the regression model, the choice of express transport operator is taken as a basis, so the analysis is carried out by comparing this service. It is possible to observe that in the case of the low-cost transport operator, the somatic reaction from the choice of the tourist transport operator is positively related, while the other somatic reactions are not significant.

Meanwhile, in the case of the tourist transport operator, a significant and negative relationship is observed concerning an increase in the somatic reaction of the Express service, so that the higher the level of somatic reaction of the Express service, the lower the probability of the tourist service option be chosen. Additionally, a significant and positive relationship can be observed with the somatic reaction of the tourist company, which implies that the higher the level of somatic reaction, the greater the possibility of being chosen in the next period.

## Discussion

The experimental results were obtained from the execution of 100 independent cycles, each of them consisting of 100 travel requests generated by the passenger agent in a sequential manner. In total, 10,000 trip requests were analyzed. It should be noted that all these travel requests were generated from a single configuration of parameters available in Table 1.

The experimental scenario considered a single passenger agent profile. Given that three travel proposals were generated for each trip request (one for each type of transport operator), the option that the passenger agent chose the proposal with the best score was always considered. The trip metrics were the same for each proposal from each transport operator. The results indicate a trend toward passengers preferring travel options provided by transport operators with a touristic profile. This suggests that passengers may prioritize factors other than cost or travel time. For example, some recent studies have shown that passengers give greater importance to factors other than cost or travel time, such as in-vehicle environment (e.g., comfortable seats and temperature), frequent and regular service, and driver's attitude^52^, vehicle condition and hygiene^53^, and the perceived vehicle and driver-related risk^54^. Additionally, the results demonstrate that express or low-cost transport operators occasionally provide successful trips. It is the global score indicator that gives flexibility to the passenger agent to select the travel proposal that balances the metrics of time, cost, and utility, all the above along with the *somaticIndex* variable.

The general experimental scenario studies an artificial map that simulates characteristics usually present in a city, such as vehicular congestion, the insecurity of certain streets, or the benefits of an area with a tourist profile. The map can be extended in terms of dimensions and characteristics by, for example, incorporating sections of high-speed highways, specifying street or neighborhood types through which it travels (university, commercial, and residential), and highlighting critical public services (hospitals, police).

Meanwhile, Eqs. (1), (2), (3), and (4) allow determining essential metrics of the passenger agent's movement on the map. A change in the characteristics of the map also entails reviewing and updating the equations that determine the travel metrics.

Equation (5) considers travel metrics and *somaticIndex* variable, which synthesizes the passenger agent's level of travel experiences with a specific transport operator. This index is updated based on the artificial somatic reaction experienced by the passenger agent when notified to replan the initially informed travel route. It should be noted that the somatic reaction equation considers a random factor, so that in the eventual presence of identical stimuli, the artificial somatic reaction in the passenger agent does not yield the same result.

The current updating mechanism of the *somaticIndex* variable gives an equivalent weight to both the record of past experience and the last somatic reaction that took place in the passenger agent (derived from the most recent travel experience with a specific transport operator). It is possible to modify the current proposal to reflect the passenger agent's travel experience. An alternative is to give more importance to the recent somatic reaction to the extent that the travel experience with a specific transport operator becomes systematically poor. Another alternative is to determine a proportion of recent somatic reactions over the total of recorded travel experiences with a specific transport operator.

The general trip request process algorithm is defined from the passenger agent's perspective, encompassing all the steps that the passenger agent requires to evaluate and select the travel proposal and to record their travel experiences. The foregoing also considers the option of being able to process a route replanning in real-time. In this work, this replanning was always considered within the experimental scenario to derive both the somatic reaction and the updating of the *somaticIndex*. The present work establishes the context in which the passenger agent should face some circumstance that would lead to some type of somatic reaction. Currently, it is accepted that the generation of memories is directly influenced by people's somatic states^55^. Thus, this accumulated travel experience is reflected in the following decisions of the passenger agent.

In the experimental scenario, the autonomous system recurrently requests to be transported considering the same origin–destination coordinate pair. This seeks to represent a passenger who needs to be transported frequently between usual places (e.g. home to work and work to home) or between places within a city that are weakly covered by the conventional public transport system (in frequency or in travel experience), or directly without coverage.

The current work seeks to reflect the experience of a user concerning his trip. In this case, the passenger agent acquires information from his previous trips (from the *somaticIndex* variable). About the User Equilibrium criterion^56^ and the present research work, it is possible to indicate that: (1) in the conformation of each route option, the information about each route segment is known during the route generation process itself (since the algorithm for path generation, presented by^51^, is a constructive algorithm in nature); (2) the passenger agent will not necessarily select the route option with the lowest cost or time; (3) the passenger agent profiles have different characteristics that differentiate them from each other.

On the other hand, mental models are analog representations that preserve the structure of the thing they represent^57^. Cognitive psychology suggests that a mental model is composed of two major components: knowledge structures (schemes) and processes for using this knowledge (mental operations)^58^. Meanwhile, emotions and somatic reactions influence human perception and decision-making^18,59^. Considering the above, the proposed artificial mental model is bio-inspired in cognitive human processes and disaggregates the different cognitive functions present in people into specialized components. The proposed artificial mental model can be further specialized, for example, by incorporating sub-components, allowing facing thematic deliberative processes based on the types of problems to be addressed, or designing sub-components that allow recording events or memories through alternative mechanisms. Likewise, the management of the affective dimension could be grouped within a specific additional layer that can be transversal to the three existing levels in the artificial mental model.

The proposed artificial mental model represents an abstract approach to the human mind. It corresponds to a static view, that is, it does not describe a flow or procedure. In this sense, the relationship between the components requires the definition of procedures that make articulated use of them. As an example, in algorithm 1 the component "Executive Process Mechanism" is used in steps 1, 2, and 4. Lines 7 to 11 represent actions that can be associated with a "Specific Solving Mechanism." Meanwhile, step 24 represents an action that can be associated with the "Somatic Engine" component, while step 25 represents an action that can be associated with the "Secondary Emotions Engine" component. For its part, line (27) is an action associable to the "Long-Term Memory" component.

Due to the specific context of the experimental scenario, the reflective level of the artificial mental model was not considered in the evaluation of the proposal. However, incorporating the reflective level would enable the passenger agent to retrospectively evaluate their experiences derived from the use of the service provided by each transport operator. This would allow the passenger agent to share their opinions derived from their perception of the service received. Consequently, the decision regarding which travel proposal to select could be based not only on travel metrics (i.e., objective dimension) and individual travel experience (i.e., individual subjective dimension) but also on the opinion of other passengers (i.e., social subjective dimension).

The purpose of the trip, preferences and psycho-emotional factors must be known, captured and modeled by the passenger agent that corresponds to the client's digital assistant (and not transferred to the operator) under a decentralized planning or assignment approach. Then, the model proposed in this article focuses on a particular aspect of user preferences. These are their psycho-emotional reasons/factors and modeled in terms of a psychosomatic factor. These factors, and therefore the proposed model, can be complemented with more rational factors for capturing preferences and valuing trips. Our proposal is in line with expanding and enriching the set of factors and measures to model customers' preferences and route selection mode in a flexible passenger transport scenario, and not replacing the factors and measures with purely psycho-emotional ones.

In relation to the distance factors of each type of street traveled, and with a view to the implementation of a mobile app or electronic platform, they can be obtained and derived from the application of questionnaires (with Likert scales) to reveal people's preferences, and thereby training or adjusting the weights and parameters of the model to the characteristics and preferences of the user.

Given the decentralized nature of service planning, different actors (passenger agents, transport operators) can pursue different objectives and therefore each one can be modeled differently and considering their own measures and variables. Operators could try to minimize the number of vehicles and balance the routes within those used vehicles, while customers could seek to satisfy their travel preferences, but not in a group mode but rather individually.

The proposed model can also be extended to passengers of private vehicles, under the scenario in which a driver asks his personal digital transportation assistant (e.g. Waze, Google Maps) which is the best route between two points, but not only considering distance, vehicle congestion, current or historical average speed of the different alternatives, but also incorporates the preferences in terms of previous personal experiences of said driver. For example, an experienced and perhaps more risky driver will not distinguish or select a street that has priority at intersections with other streets, compared to another where he has to give up preferences. However, a more novice or new driver will prefer easier, safer and less demanding routes in terms of driving skills (e.g. calculating distances to cross an intersection or pass another vehicle). Other factors that can affect drivers' preferences may be the number of curves, the slope of the street, the general movement of other vehicles, buses and even pedestrians that may be crossed. In this sense, good and bad previous experiences will clearly influence a driver's choice of streets and routes. And in this sense, the proposed model seeks to complement current preference models so that they can incorporate these conditions due to past experiences.

This research work presents an autonomous decision-making system that represents a human passenger under a flexible passenger transport modality, specifically, in a case of a passenger who makes a frequent trip between an origin and a destination. Unlike current cases in the industry, this research work explores the case where the passenger can have as a client a trip profile with memory, differentiated from other clients, and can receive more than one trip proposal for the same trip request, according to the different conditions that the passenger is looking for. The current proposal extends the service typically seen on flexible transportation mobile platforms, where people are limited in their options for choosing routes and in the automated recording of their travel experiences. In this sense, affective artificial intelligence offers a reasonable alternative to extend current decision models and develop sensitive business models with human life experience. Indeed, it is necessary to deepen the analysis on the incorporation of AI within technologies to support the movement of people, in such a way as to explore business models that, while remaining viable, give greater importance to human factors and flexible travel conditions.

In the future, technological systems accompanying people in their daily lives will not only be oriented toward recommending decisions for said people (e.g., which product to buy, what urban travel service to hire, and what tourist destination to visit) but also possess the capability to fully and autonomously carry out decision-making processes. For individuals to be able to fully delegate their decisions to autonomous technological systems, these systems must not only possess the computing capacity to operate under big data scenarios, cloud environments, and real-time constraints but also encompass the criteria and variables that represent both human thinking and feeling in their decision-making. Traditional technological approaches and decision models, often assuming full rationality in human decision-making, will progressively give way to new forms and approaches of decision systems and models that embrace a greater richness in terms of decision criteria. These criteria will be defined based on the complexity of the modern world, where the purchase of the most reliable product, the selection of the cheapest travel route, and the choice of the most popular tourist destination are not always the only determining factors. People are essentially rational-emotional beings, and therefore, the nature of their decisions must be reflected in autonomous systems that aspire to be representative of human decision-making.

## Conclusion

This work analyzed the implementation of an artificial mechanism bio-inspired in a biological somatic marker, which enables a passenger agent to react to changes in the service and retain those reactions as memories for future decisions. An artificial mental model was designed to incorporate both the representation of cognitive components and the presence of artificial somatic reactions of a passenger agent. The passenger agent was implemented as an autonomous decision-making system, where both the choice of the transport operator and the evaluation of the service received were fully delegated to the system. Hence, the evaluation of the service experience was based not only on rational aspects, such as the cost of the trip, but also on subjective aspects related to the level of satisfaction derived from the passenger's experience.

Our artificial mental model presents three levels and two cognitive layers (whereas Minsky's model^50^ suggested seven levels). The algorithm of the present research work, specifically in line (5), assumes that another additional algorithm operates generating routes (presented by^51^). Meanwhile, the somatic marker hypothesis presented by Damasio^18^, was not applied by himself at an artificial level. In this sense, our proposal designs a mechanism bio-inspired in Damasio's proposal but in artificial terms.

One limitation of this work is that it only considers the participation of a single passenger agent as an entity requesting transport services. Another limitation is that real-time route replanning does not distinguish or report the cause, even though different types of causes could lead to different types of artificial somatic reactions. Moreover, the experimental results were carried out using a single configuration of parameters. Another limitation corresponds to the use of synthetic data, along with a general definition of values for simulation parameters (Supplementry file).

A future line of work corresponds to calibrating the simulation parameters from empirical data, intending to reflect different types of travel profiles from real passenger profiles. Likewise, to incorporate different magnitudes of reactions for the *somaticReaction* variable, according to the variations in the travel proposals received.

Another area of future research could focus on exploring alternative mechanisms for updating the *somaticIndex* variable. As mentioned, the current mechanism recognizes the concept of somatic reward or punishment. However, it is worth considering the possibility of analyzing other alternative mechanisms that allow for flexible variations in the magnitude of the reward or punishment. This would provide a more adaptable approach to evaluating the impact of different experiences. Another line of future work involves expanding the number of passenger agents, in such a way that the individual evaluation model of the travel proposals received can consider with some degree of influence the travel experience of other passengers. Furthermore, future studies can explore the incorporation of compensatory mechanisms by transport operators who have demonstrated subpar performance in providing their service.

### Supplementary Information

Supplementary Information.

## Data Availability

All data generated and analyzed during this study are included in this published article.
